# Efficient Recovery of Lithium Cobaltate from Spent Lithium-Ion Batteries for Oxygen Evolution Reaction

**DOI:** 10.3390/nano11123343

**Published:** 2021-12-09

**Authors:** Ayesha Arif, Ming Xu, Jamshaid Rashid, Chaudry Sajed Saraj, Wei Li, Bilal Akram, Binbin Hu

**Affiliations:** 1 Department of Environmental Science, Faculty of Biological Sciences, Quaid-I-Azam University, Islamabad 45320, Pakistan; a_ayesha113@outlook.com; 2 BNU-HKUST Laboratory of Green Innovation, Advanced Institute of Natural Sciences, Beijing Normal University at Zhuhai, Zhuhai 519087, China; 3 College of Environment and Planning, Henan University, Kaifeng 475004, China; 4 Key Laboratory of Geospatial Technology for the Middle and Lower Yellow River Regions, Ministry of Education, Henan University, Kaifeng 475004, China; 5 GPL, State Key Laboratory of Applied Optics, Changchun Institute of Optics, Fine Mechanics and Physics, Chinese Academy of Sciences, Changchun 130033, China; sajedsaraj@ciomp.ac.cn (C.S.S.); weili1@ciomp.ac.cn (W.L.); 6 Department of Chemistry, Tsinghua University, Beijing 100084, China; bilalakram626@gmail.com; 7 Key Laboratory for Special Functional Materials of Ministry of Education, National & Local Joint Engineering Research Centre for High-Efficiency Display and Lighting Technology, School of Materials and Engineering, Collaborative Innovation Centre of Nano Functional Materials and Applications, Henan University, Kaifeng 475004, China; hbb@henu.edu.cn

**Keywords:** lemon peel extracts, lithium-ion batteries, oxygen evolution reaction, renewable energy, waste management

## Abstract

Owing to technological advancements and the ever-increasing population, the search for renewable energy resources has increased. One such attempt at finding effective renewable energy is recycling of lithium-ion batteries and using the recycled material as an electrocatalyst for the oxygen evolution reaction (OER) step in water splitting reactions. In electrocatalysis, the OER plays a crucial role and several electrocatalysts have been investigated to improve the efficiency of O_2_ gas evolution. Present research involves the use of citric acid coupled with lemon peel extracts for efficient recovery of lithium cobaltate from waste lithium-ion batteries and subsequent use of the recovered cathode material for OER in water splitting. Optimum recovery was achieved at 90 °C within 3 h of treatment with 1.5 M citric acid and 1.5% extract volume. The consequent electrode materials were calcined at 600, 700 and 800 °C and compared to the untreated waste material calcined at 600 °C for OER activity. The treated material recovered and calcined at 600 °C was the best among all of the samples for OER activity. Its average particle size was estimated to be within the 20–100 nm range and required a low overpotential of 0.55 V vs. RHE for the current density to reach 10 mA/cm^2^ with a Tafel value of 128 mV/dec.

## 1. Introduction

With the advancements in technology and the growing population, continuously degrading the environment and increasing the demand of energy have become mankind’s major concerns. The use of renewable sources for energy conversion has been deemed an inspiring solution to reduce the dependance on the unsustainable exploitation of fossil fuels, which is also a major cause of growing pollution in the environment. Some of the major renewable energy resources include fuel cells and metal air batteries. Oxygen evolution reactions (OER) are the fundamental reactions for the above-mentioned renewable systems [1]. In OERs, the molecular oxygen is produced through a multistep four-electron oxidation reaction, thus considered kinetically sluggish [2]. Therefore, the need for an effective and efficient electrocatalyst comes forward to reduce the overpotential and to accelerate the reaction, thus increasing the energy conversion efficiency. Generally, precious metals and transition metals such as Pt, Ir, Co, Mn, and Ru, etc., are needed to promote OERs. Ru and Ir, to this day, are still considered as the most efficient electrocatalysts for OERs as they show a relatively low overpotential and a Tafel slope [3]. Another case is the use of transition metal oxides as OER electrocatalysts due to their multiple oxidation states, low-cost and good corrosion resistance [4]. NiO, for example, is receiving huge attention in the field of electrocatalysis. Many studies have reported the fabrication of higher oxidation states of Ni, which have proven to be very active for OERs. Fominykh and co-workers fabricated NiO NPs by solvothermal reactions, in which an Ni^3+^ state was formed on the surface and obtained excellent results [5]. The synergistic effect of transition metals has also been wildly studied. Lankouf and colleagues studied the impact of adding Mn to the cubic Co_3_O_4_ and obtained increased electrocatalytic activity with a current density of 10 mA cm^−2^ at a relatively low overpotential of 327 mV [6]. Another study demonstrated the use of ultra-thin Co nanosheets coupled with N-doped carbon plate, which possessed a high specific surface area of 446.49 m^2^ g^−1^, which resulted in its efficient performance with an overpotential of 278 mV at 10 mA cm^−2^ [7]. Zhang et. al., fabricated ZnO/Co_3_O_4_ core-shell nanorods on Ni foil, which exhibited excellent electrochemical performance for OERs, with an overpotential of 294 mV at 10 mA cm^−^^2^, a Tafel slope of 49 mV dec^−^^1^, and excellent stability [8]. However, the high costs and low reserves of these metals limits their usage at a commercial level. In this respect, scientists all over the world are making efforts to develop novel electrocatalysts to replace these metals completely or partially or at least lessen their costs. One of such attempts is the use of cathode material used in lithium-ion batteries (LIBs) for OERs. Lithium-ion batteries have been largely employed in various devices such as laptops, video cameras, mobile phones and other portable appliances [9], electric vehicles and photovoltaic cells [10], owing to their characteristics such as long-life cycle, light weight, high working voltage, no memory effect, high energy density, light weight, small size and low self-discharge rate [11]. The cathode of lithium-ion batteries is made up of lithium-transition metal oxides [12], which have been tested for the OER activities [13,14]. Chen and co-workers developed a simple method to convert the recycled LiCoO_2_ into an electrocatalyst for OERs. They cycled the battery several times and discovered that after cycling for 500 cycles, the recycled LiCoO_2_ can deliver a current density of 9.68 mA cm^−2^ at 1.65 V [15]. Lu and colleagues demonstrated a method for the electrochemical lithium tuning of catalytic materials in organic electrolytes to enhance the catalytic activity in an aqueous solution. By continuously extracting lithium ions out of LiCoO_2_, the catalytic activity can be improved [16]. However, the same problem is noticeable in this case as well, i.e., short reservoirs. The components of lithium-ion batteries are considered “critical minerals” by the U.S. government due to their short reservoirs and fluctuating prices [17]. For a decade, a debate on the importance of recycling of Li-ion batteries has been ongoing. Cobalt is mined majorly in African countries such as Congo and Zambia, as well as other vital countries, including Australia, Brazil, Cuba, Canada, Russia, Madagascar, and China. The volatile pricing of cobalt due to supply demand contrast and ongoing global issues is wreaking havoc for both manufacturers and miners [18,19]. Although lithium reserves of 53.5 million tons worldwide may seem to last for a century, not all the resources are recoverable, and many require extremely high costs, such as Li recovery from brine and sea water etc. [20]. The recoverable resources of Li are limited to the Li-triangle (China, Chile, Argentina, and Bolivia). Due to its increasing demands, the price of lithium is also rising [21,22,23]. The hazards of waste LIBs are an additional factor to be considered while discussing the need to recycle theses batteries [23]. Previous studies employed methods such as pyrometallurgy, which involves the reduction of metals by heating of the waste LIBs at a very high temperature [24,25], or hydrometallurgical processes [26,27], for the recovery of cathode material from waste LIBs. However, all of these methods have drawbacks. The hydrometallurgical process, for instance, can salvage and purify battery materials with a high yield; however, the unjustified consumption of alkalis and acids causes secondary pollution and the corrosion of equipment [28]. On the contrary, pyrometallurgy has the disadvantages of high temperature requirements (above 1300 °C), which results in high energy consumption, along with high pollution and low extraction efficiency of lithium. The present study reports, for the first time, the use of environmentally friendly lemon peel extract and citric acid for the recovery of Li and Co from spent LIBs due to their superior leaching performance, low toxic emissions, easy natural degradation, and potentially low price. Recycled LiCoO_2_ was calcined at different temperatures, characterized by X-ray photoelectron spectroscopy (XPS), X-ray diffraction (XRD), scanning electron microscopy (SEM), and transmission electron microscopy (TEM), and subsequently tested and compared for OER performance.

## 2. Experimental Section

### 2.1. Materials and Instruments

The glassware utilized in the experimental procedure included beakers (1000 mL and 250 mL), measuring cylinders, spatulas, pipettes, Whattman filter paper, and crucibles. The glassware was made up of either Pyrex or borosilicate glass. Citric acid (C_6_H_8_O_7_) was used as a leaching agent. Sodium carbonate (Na_2_CO_3_) and oxalic acid (C_2_H_2_O_4_) were used as precipitating agents. All of the chemical reagents used were purchased from Sigma Aldrich (St. Louis, MO, USA). All solutions were prepared or diluted by deionized water. Spent lithium ion (mobile phone) batteries were collected from various colleagues. The lemon peel extract was prepared by using lemons bought from the local market. Peel extract was made by drying the lemon peel from the used lemons and then grinding the peel into powder. The powder was then heated at 100 °C for 1 h in deionized water. The solution was filtered and stored in a refrigerator for future use. To weigh the chemicals, a Shimadzu ATY244 microbalancer (Shimadzu, Kyoto, Japan) was used. An 8000 Adwa pH-meter (Adwa, Romania) was used for the measurement of the pH of the chemical and reaction mixtures. A muffle furnace was utilized for calcination during the preparation of lithium metal oxide. To note the changes in temperature, a thermometer was used. An Atomic Absorption Spectrophotometer (AAS), (AA-7000 by Shimadzu, Kyoto, Japan) and Flame Photometer (Model 360 Flame Photometer by Sherwood, Cambridge, UK) were used for quantitative analysis of the cobalt and lithium recovery by leaching.

### 2.2. Metal Recovery and Sample Preparation

**Figure 1:**
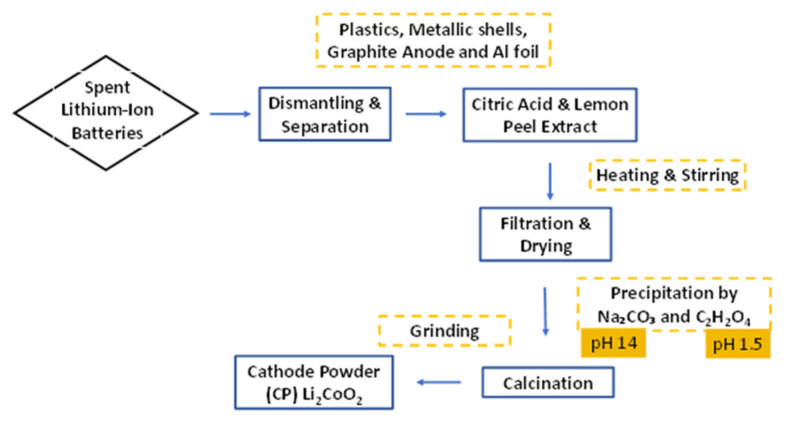
Schematic diagram for the leaching and recycling of an LIB cathode.

The positive and negative terminals of batteries, if handled irresponsibly, will ignite. Hence, there is a need to discharge them first. For this purpose, the batteries in this experiment were discharged by being connected to a 100 ohm load and the batteries were steadily discharged to a safe voltage of 1.0 volts. Then, the batteries were manually dismantled. Next, the binder (PVDF) was removed to separate the cathode material from the Al foil (current collector) by dissolving the foil in DMSO. The obtained powder was dried in an oven for 1 h at 100 °C. Then, the resultant powder was heated at 600 °C in a furnace to get rid of the carbon and binder or any other organic matter present. The powder was then crushed and grounded manually. The leaching experiments were conducted in a 250 mL flask, placed on a hot plate to control the temperature. A thermometer was used to constantly check the temperature of the reaction mixture. A magnetic stirrer was used to stir the material uniformly. The flask was then covered with aluminum foil to avoid the unnecessary evaporation of the reaction mixture. A predetermined quantity of the cathode material (2 g) was accurately measured, and citric acid and peel extract in different concentrations were added to the flask. During the experiments, the stirring was kept constant to 300 rpm. The amount of lemon peel extract and the acid concentration were varied to obtain the optimal efficiency. Similarly, the temperature and time were varied to achieve the optimum conditions. The periodically collected samples were filtered using a syringe filter (0.2 µm). As a result, a black residue, and a filtrate of different shades of pink were obtained. The filtrates were estimated for Co and Li ions using an atomic absorption spectrometer (AAS), and the leaching efficiency of lemon peel was measured using the following formula [29]:

$$y=\frac{Cw,t\times V}{mw}\times 100\%$$

where: *y* is the leaching efficiency in %, *C_w,t_* denotes the concentration (g/L) of metal W (Li and Co) at a given time, *V* is volume of the leachant (L), and *m* denotes the mass of metal in the cathode scrape (g), which was calculated by dissolving the cathode material (approximately 2 g) completely in aqua regia, and AAS was used to reveal the mass of Li and Co in the cathode scrape. A diagrammatic representation of the recovery process is provided in Figure 1.

### 2.3. Reductive Leaching

Citric acid falls under the category of polyprotic acids, which can dissociate and give out more than two hydrogen ions in an aqueous solution [30]. As a result, the following reaction takes place when LiMO_2_ is placed in the reaction mixture:

H_3_Cit + LiMO_2_
**→** Li^+^(aq) + H_2_Cit^−^(aq) + M^+^(aq) + H^+^ + O_2_(g) (1)

However, the transition metals in the layered structure are difficult to remove from the lattice. This situation can be improved by adding a reducing agent, as shown in previous studies. However, the addition of a reducing agent must not make the process economically and/or environmentally unfeasible. In the present study, lemon peel extract was used as a source of reducing agent (ascorbic acid), as it has more ascorbic acid (Vit C) than its juice [31,32]. Ascorbic acid has good reducing ability, as it can provide two electrons and can be oxidized two times to the stable dehydro-ascorbic acid (C_6_H_6_O_6_) [33]. Furthermore, it is also able to provide hydrogen ions for the leaching process, hence efficiently speeding up the reaction efficiently [34]. After the leaching of metals (lithium and cobalt), the metals were removed and recovered by adding sodium carbonate (2 mol L^−1^) and oxalic acid (1 mol L^−1^) in their precipitate forms [35]. Precipitation reactions were carried out in a 250 mL flask on hot plate. A magnetic stirrer was used for the stirring of the reaction mixture, and Al foil was used to cover the flask to avoid evaporation of the reaction mixture. The recycled material (precipitates of lithium carbonate and cobalt oxalate) was then combined and calcined at different temperatures to harvest the active cathode material. The precursors (precipitates obtained by precipitation) were mixed at a ratio of 1.05/1 (Li/Co) heated at 600 °C (CP3), 700 °C (CP1) and 800 °C (CP2) in a muffle furnace.

### 2.4. Electrode Preparation

A BioLogic VMP3 multichannel workstation with a three-electrode system was utilized for electrochemical measurements, where a Pt wire, a catalyst-loaded carbon cloth electrode, and a saturated calomel electrode (SCE) were used as a counter and working and reference electrodes, respectively. Aqueous solutions of 1M KOH were used as alkaline electrolytes for the electrochemical measurements. LSV curves were measured by sweeping voltage in the range of −0.2 to −1.6 V vs. the SCE electrode with the a scan rate of 10 mV·s^−1^. The expression $E_{RHE}=E_{SCE}+E_{SCE}^{0}+0.0592\mathrm{pH}$, where $E_{SCE}^{0}=0.242\;V$, was used to translate V vs. SCE to V vs. the reverse hydrogen electrode (RHE). Working electrodes were first pre-stabilized in the electrolyte solution using 30 scans of cyclic voltammetry at 20 mV·s^−1^ before performing linear sweep voltammetry measurements. Electrochemical impedance spectra (EIS) were recorded with the biasing of the working electrode at −1.4 V (HER) and 0.5 V (OER) (vs. SCE) and superimposing a small alternating voltage of 10 mV over the frequency range of 0.01 Hz to 1 MHz. The CV curves were further measured in the non-Faradaic region of potential from 1.091 to 1.191 V (vs. RHE) with different scan rates (from 10 to 120 mV·s^−1^) to estimate the double layer capacitance (Cdl).

### 2.5. Analytical Methods

The crystalline nature of the recovered material was characterized by X-ray diffraction (XRD, Bruker AXS-D8, Billerica, MA, USA), which was carried out using Cu-Kα (λ = 1.5406 nm) as a source of radiation and secondary monochromator in the range 2θ from 10 to 80°. The surface morphologies of the samples were explored by SEM (Hitachi S-4800, Tokyo, Japan at an operating voltage of 25 kV, as well as by JEOL-2100 TEM). For the verification of chemical or elemental composition of the materials Energy Dispersive X-ray Analysis (EDAX) were performed using the Omicron system (Al Kα 1486.7 eV X-ray source operated at 15 KeV). For a better insight of the OER activities X-ray Photoelectron spectroscopy was performed using the Omicron system (Al Kα 1486.7 eV X-ray source operated at 15 KeV) at constant analyzer energy (CAE) = 100 eV for survey scans and 20 eV for detailed scans. A binding energy of 284.8 eV of C1-s was used for calibration.

## 3. Results and Discussion

### 3.1. Metal Recovery

The proposed reaction for the reductive leaching process can be represented as follows [36]:

6H_3_Cit + 2LiCoO_2_ → 2Li^+^(aq) + 6H_2_Cit^−^(aq) +2 Co^2+^(aq) + 6H^+^ + 2O_2_(g) (2)

6H_2_Cit^−^(aq) + 2LiCoO_2_ → 2Li^+^(aq) + 2Co^2+^ + 6H^+^ + 6HCit^−2^(aq) + 2O_2_(g) (3)

6HCit^−2^(aq) + 2LiCoO_2 →_ 2Li^+^(aq) + 2Co^2+^ + H^+^ + 6Cit^−3^(aq) + 2O_2_(g) (4)

The efficiency of the method utilized for the recovery of crucial metals, i.e., Li and Co, from waste lithium-ion batteries was evaluated by repeating the experiments three times under same conditions, and their average was taken. The influencing parameters, such as temperature, time, acid concentration, and peel volume, were studied. During the experiments, stirring was kept constant at 300 rpm. The intermittently collected samples were filtered using a syringe filter (0.2 µm) and assessed for Co and Li ions using an AAS.

#### 3.1.1. Temperature and Time

**Figure 2:**
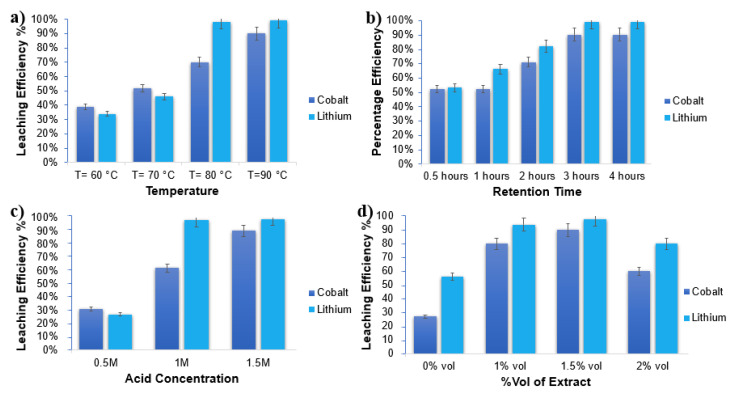
(**a**) Impact of temperature on leaching efficiency. (**b**) Impact of retention time on leaching efficiency. (**c**) Impact on the acid concentration of leaching efficiency. (**d**) Impact of extract vol% on leaching efficiency.

The impact of temperature and retention time was studied, as portrayed in Figure 2. It was observed that leaching of both lithium and cobalt gradually increased when the temperature raised from 70 °C, then to 80 °C and 90 °C (Figure 2a). The leaching efficiency was noted to be 39% for Co, 34% for Li, 52% for Co, 46% for Li, and 70% for Co, and 98% for Li at temperatures of 60 °C, 70 °C, and 80 °C, respectively. The maximum leaching efficiency was obtained at a 90 °C temperature when the mixture was heated for 3 h. At this stage, 90% of Co and 99% of Li was leached. This increase in leaching efficiency is attributed to the fact that a higher temperature can raise the speed of the molecular motion and increase the energy of a particle’s collisions. No further tests were conducted with an increased temperature, since a marginal increase in leaching efficiency may prove to be costly due to the significant increase in energy consumption by raising the temperature from 90 to 100 or 110 °C. A balanced temperature of 90 °C was considered as the optimal temperature for the remaining experiments. Figure 2b depicts 3 h to be the optimum time for the metals (lithium and cobalt) to reach maximum dissolution, indicating that the leaching equilibrium had been reached. Hence, 3 h was taken as the optimal leaching time.

#### 3.1.2. Acid Concentration and Extract Volume

The impact of the citric acid concentration was also investigated under an optimum condition of 1.5% by volume of extract, at a 90 °C temperature for 3 h of the experiment. As shown in Figure 2c, an insignificant increase in leaching efficiency was observed at an acid strength of 0.5 M, i.e., 31% and 27% for cobalt and lithium, respectively. However, with an increase in acid strength, the leaching efficiency improved significantly. At a 1.5 M acid concentration, 90% cobalt and 99% lithium were recovered. Experiments were also conducted with a varied amount of lemon peel extract to check the impact of the concentration of peel extract on the leaching efficiency of lithium and cobalt. Citric acid alone, as shown in the Figure 2d, had no significant leaching activity. Cobalt, showed leaching efficiency of only 27% in 3 h and 90 °C temperature. However, the maximum leaching efficiency was observed when the volume of the extract was increased. The maximum leaching efficiency of 98% and 90% for lithium and cobalt, respectively, was obtained with extract % volume of 1.5% at a 90 °C temperature and after 3 h of the experiment. However, a further increase in peel extract resulted in a decrease in the leaching efficiency for both lithium and cobalt. Lemon peel extract, as a reducing agent, was used, together with citric acid, as a complexing agent. The reducing agents in the lemon peel extract may have disturbed the lattice, leading to the leaching of Co II, as well as Li^+^ ions, through complexation. Since citrate is a strong complexing agent, both Co and Li ions were anticipated as citrate complexes. As the dissolution proceeded, the initially black solution turned dark pink. All of the parameters provided results in accordance with many of the previous studies. For example, Nayaka [37] used ascorbic acid, along with citric acid, and achieved the maximum efficiency (100% in the case of Li and 80% in the case of Co) in 6 h. The present study reached the maximum efficiency within 3 h. The cause may have been the presence of phenolics and saccharides in the lemon peel extract that aided in speeding up the reaction and helped along the reduction in Co in a lesser time. After 3 h of the reaction, there was no improvement in the leaching efficiency. Some studies, however, have shown some discrepancies. Small contrasts of the obtained results related to the metal (Li and Co) yield compared to those in the literature were also indicated. Chen and co-workers proposed a hydrometallurgical process and obtained leaching efficiencies as high as 95%, for Co and 99% for Li using 2 mol L^−1^ of citric acid, at 80 °C, a 90 min retention time, and 2 vol% H_2_O_2_ [38]. Similarly, Yu et al., in a similar study obtained 99% recovery rate under conditions, such as 1.0 M citric acid, and 8% H_2_O_2_ at 70 °C in 70 min [39]. The different experimental and preconditions resulted in slight differences in the results.

### 3.2. Material Characterization

**Figure 3:**
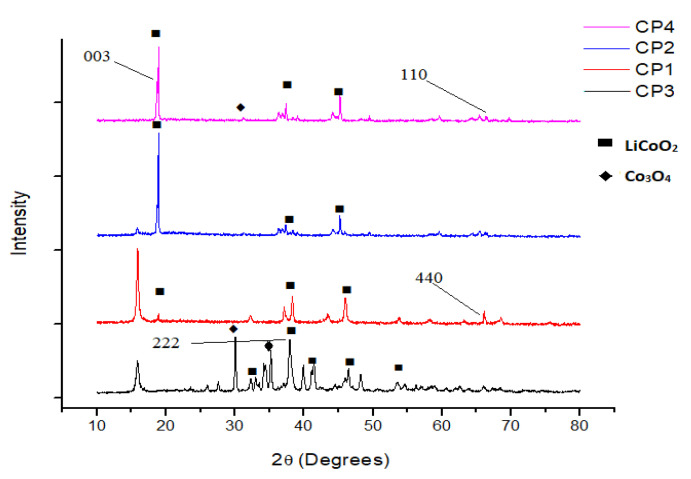
X-ray diffractogram of CP4 (waste material after calcination at 600 °C), CP2 (recovered material, calcined at 800 °C); CP1 (Recovered material, calcined at 700 °C), and recovered material, (calcined at 600 °C).

Figure 3 shows the impact of the calcination temperature on the crystallographic structure of LiCoO_2_. It can be clearly observed that samples CP4 (waste cathode material calcined at 600 °C) and CP2 (recovered material calcined at 800 °C) indeed constituted indeed of active cathode materials as matched with the literature [40,41,42], with a hexagonal α-NaFeO_2_ layered structure with an R-3m symmetry (JPCDS card No. 50-0653). The waste cathode material however showed peaks of Co_3_O_4_ (JCPDS card No. 073-1701), identifying it as Co_3_O_4_ with a cubic structure [43]. The presence of Co_3_O_4_ in sample CP4 might be due to the solid reaction that occurs during the charge–discharge cycles [44]. When the calcination temperature was lowered a crystallographic structure was obtained i.e., sample CP1 (recovered material calcined at 700 °C) and CP3 (recovered material calcined at 600 °C) showed some peaks that matched the Fd3m-type space group (JPCDS card No. 74-1631) with the spinel setting for example, the disappearance of a 003 peak in both samples, the appearance of a 440 peak in CP1 and CP3 in place of original 110, and the appearance of a 222 peak in CP3 [41,45,46]. Sample CP3 also contained phases of Co_3_O_4._ The presence of Co_3_O_4_ in the sample CP3 might have resulted from the fact that that incomplete lithiation occurs at low temperatures, as shown in previous studies [46]. The organic impurities shown as the addition peaks near 15° (2θ) in CP3 and CP1 disappeared in CP2 when the temperature was raised to 800 °C.

To estimate the chemical compositions of the synthesized powders, EDAX analysis was performed. The main elements as proven by the literature, included Li, Co, and O_2_, with the presence of other metals as well. The presence of Ni and Mn can be understood by the fact that they are present in the chemical composition of battery active materials, as proven by the literature. These metals not only aid in decreasing the price of the batteries, but also provide stability to the LiCoO_2_ structure [47]. A small amount of Zn and Cu can be attributed to the inaccurate manual processing, for example contamination from the dismantling of the steel casing. Furthermore, it should be highlighted that the research material came from spent batteries collected from various sources, often kept in unfitting conditions that cause them to be spoiled, which can add little bits of contamination. Since EDAX is not able to detect elements with atomic numbers less than 3, Li was not detected by it. The presence of Li and the formation of LiCoO_2_ was confirmed by XRD and XPS. The BET surface area, pore size and pore volume were also measured for the samples and the results are provided in Table 1. According to the BET results, sample CP1 had the highest surface area and pore size, while the used cathode material (CP4) had the minimum.

**Figure 4:**
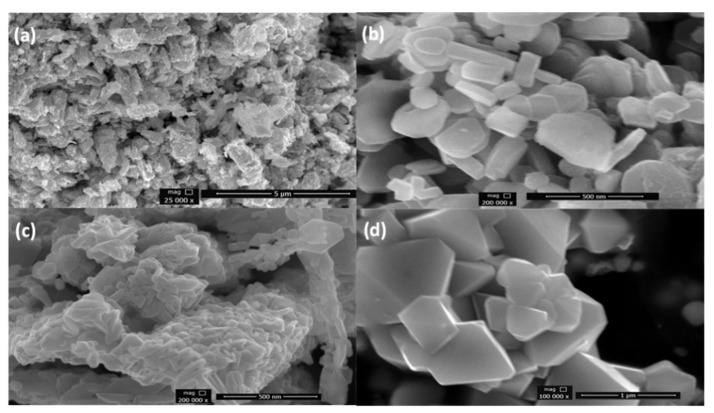
SEM results for the samples. (**a**) CP1 calcined at 700 °C after leaching, (**b**) CP2 calcined at 800 °C after leaching, (**c**) CP3 calcined at 600 °C after leaching, and (**d**) CP4 unprocessed waste cathode material calcined at 600 °C.

The powder morphologies were observed by SEM as presented in Figure 4. The SEM micrographs of the LiCoO_2_ catalysts revealed small crystallites of nanometric diameters. These particles were identified as LiCoO_2_ crystals based on the energy dispersive X-ray diffra ction studies. The SEM results of all samples showed semi-disc to rod-like shapes. Differences in the morphologies of all of the samples were observed. CP4 (Figure 4d) (waste cathode material, calcined at 600 °C) and CP2 (sample after leaching calcined at 800 °C) (Figure 4b) showed similar morphologies, i.e., no agglomeration, and well-defined particles. The other two samples showed irregularities and agglomeration. CP1 (Figure 4a), in contrast to the rest of the samples, showed a rough surface and more agglomeration as the particles can be seen clustered together. These results are in accordance with the findings of Li and colleagues [48], where the smallest size is of the samples prepared at 600 °C. Since the size of the CP3 (Figure 4c) particles was smallest among all of the samples, it had most agglomeration as well, since smaller sized particles provided more surface tension [49]. Consequently, CP2 (800 °C) had the least agglomeration. Hence, it can be concluded that the leaching has impacted the size of the LiCoO_2_ particles.

**Figure 5:**
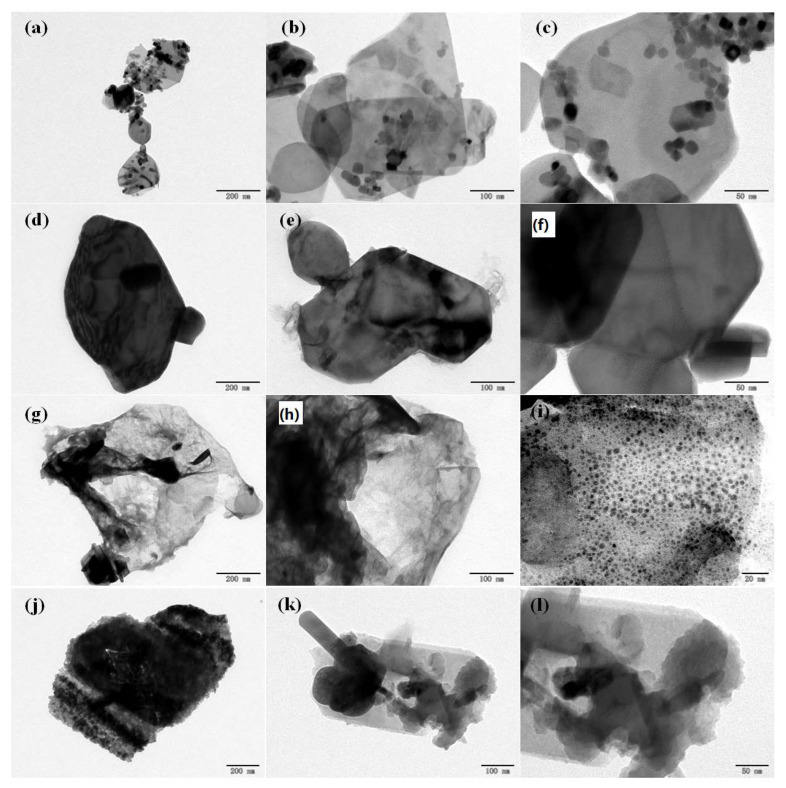
TEM results of all samples at different resolutions. (**a**–**c**) CP1 calcined at 700 °C after leaching; (**d**–**f**) CP2 calcined at 800 °C after leaching; (**g**–**i**) CP3 calcined at 600 °C after leaching; (**j**–**l**) CP4 unprocessed waste cathode material calcined at 600 °C.

The TEM results shown in Figure 5 complement the SEM findings and reveal quite similar particle size distributions. The CP1 (Figure 5a–c) showed a small particle size (within the range of 50–100 nm) while CP2 (Figure 5d–f) showed larger particle sizes (up to 200 nm). Sample CP3 (Figure 5g–i) showed extremely small particles (up to 20 nm) in diameter.

### 3.3. Electrocatalytic Performance

**Figure 6:**
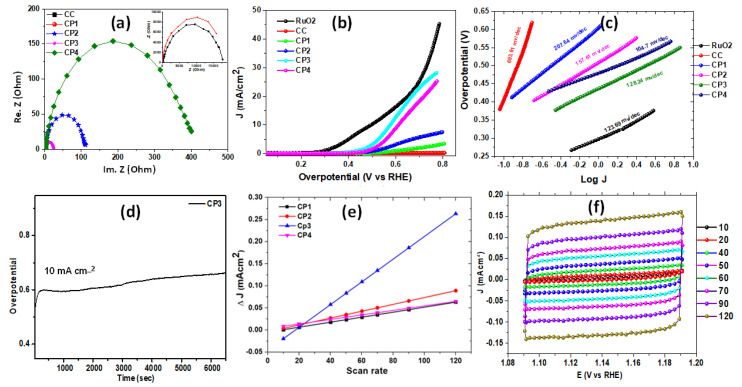
Oxygen evolution activities of the samples. (**a**) Nyquist plot; (**b**) polarization curve depicting the overpotential vs. current density of all samples; (**c**) Tafel plot; (**d**) stability curve of sample; CP3 (**e**) plot of the scan rate and current density; (**f**) CV curves for Cdl at different scan rates.

The prepared material from the recovered waste cathode of lithium-ion batteries using citric acid coupled with lemon peel extracts and the original waste material after calcination at 600 °C were tested for the OERs reactions. The OER catalytic activity of LiCoO_2_ (waste cathode material, and cathode material prepared at 600, 700 and 800 °C) is shown in the Figure 6b). All of the samples showed low overpotential. However, only CP4 and CP3 exhibited a rapid acceleration in current at low overpotential. A low overpotential (0.55 V and 0.6 V vs. RHE, respectively) was required for the current density to reach 10 mA/cm^2^. This finding contradicts with the findings of a study conducted on high temperature LIB cathode material by Pegoretti and colleagues, where high-temperature (800 °C) LIB cathode material showed an OER activity at a lower potential [50]. The difference in activity may lie in the presence of separate Co_3_O_4_ in samples CP4 and CP3 (as previously shown in the XRDs of both samples), i.e., delithiation. It has been previously proven that delithiated LiCoO_2_ has more electrochemical active sites as compared to lithiated LiCoO_2_ [16]. The Tafel slope and Rct values were obtained to evaluate the catalytic performance of a catalyst towards an OER. The Tafel slope (Figure 6c) of CP4 and CP3 showed small values of 104.7 mV/dec and 128.24 mV/dec compared to the rest of the samples under similar conditions. This slight difference indicates the similar reaction kinetics, and the smaller values of CP4 and CP3 indicates more favorable OER performance than the rest of the samples. The values of the CP4 and CP3 slopes indicates a favorable OER performance than the rest of the samples, as the smaller values are indicative of a higher electron conductivity and charge transfer process. To support the OER activity of the samples further, EIS (Nyquist real Z’ vs. imaginative Z”) was carried out on the samples. The EIS (Figure 6a) results manifested a semi-circle with a smaller diameter for CP3, which indicates the lower charge transfer resistance compared to other samples. CP4, despite its low onset potential, showed a comparatively high charge transfer resistance (R_ct_). Figure 6a shows that the R_ct_ value for the CP1, CP2, CP3, and CP4 electrocatalysts are 17,802 Ω, 111.4 Ω, 24.99 Ω, and 401.54 Ω, respectively. The C_dl_ values (Figure 6f) extracted from the CV curves recorded in the non-Faradaic region (1.09 to 1.191 V) at different scan rates were estimated (Figure 6f) to explore the intrinsic activities of the samples. Sample CP3, displayed a significantly higher C_dl_ value (2.57 mF/cm^2^) over CP1 (0.56 mF/cm^2^) and CP2 (0.77 mF/cm^2^), and CP4 (0.57 mF/cm^2^). The larger C_dl_ indicated that CP3 had a significantly increased number of exposed active sites and a higher efficient mass and charge transport capability [51]. Furthermore, as demonstrated in Figure 6e, CP3 had a larger current density at the same scan rate compared to the rest of the samples. The long-term electrochemical stability of the sample CP3 was also tested at a static current of 10 mA cm^−2^. CP4 proved to be unstable. The stability curve of CP3 (Figure 6d) showed an increase in the current density due to activation process and production of high oxidation intermediates and then became stable for 6500 s. Continuous gas evolution was observed during the stability measurement which dissipated rapidly into the electrolyte. The very small change in the potential during 6000 s indicates good durability of the CP3 OER electrocatalyst in the alkaline medium.

**Figure 7:**
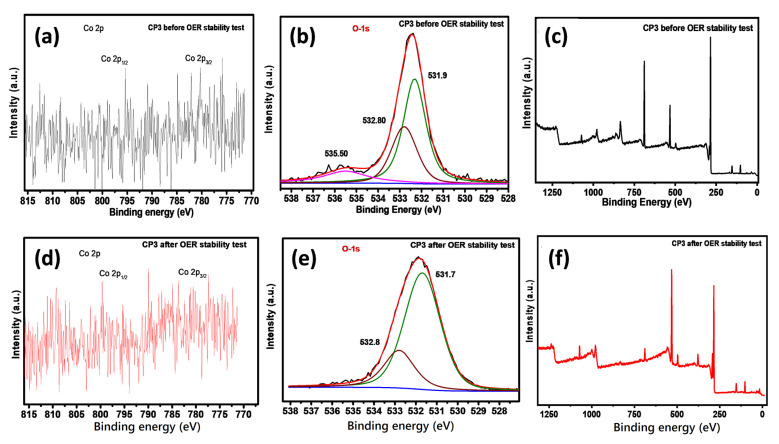
XPS spectra of Co 2p and O 1s in sample CP3; (**a**) XPS spectra representing the oxidation state of Co 2p before OER; (**b**) resolved XPS spectra representing the oxidation state of O 1s before OER; (**c**) Survey scan of CP3 before OER stability Test; (**d**) XPS spectra representing the oxidation state of Co 2p after OER; (**e**) resolved XPS representing oxidation state of the O 1s after OER; (**f**) survey scan of CP3 after OER stability test.

To dig deeper into the electrocatalytic performance of the catalyst, X-ray photoelectron spectroscopy (XPS) was employed on CP3, as it showed the best OER activity, to explore the surface composition and oxidation states of the electrocatalysts. The XPS results corroborated the findings of the XRD and EDAX analyses. As suggested by previous studies [52,53], the transition-metal-based materials exhibited excellent OER properties. The reason involves the existence of cations with mixed valence states. Furthermore, the weaker binding between cations leads to a flexible electronic configuration. This then leads to a higher conductivity and catalytic activities. Studies have revealed that, theoretically, the oxygen evolution reactions activities are vastly linked to the electronic structures of the metals, including their oxidation states. To illustrate the electronic configuration of our catalysts and their oxidation states, C 1 s peak at 284.8 eV was used as a reference for calibrating the binding energies. The binding energy values of all of the metals present in the sample showed that the Co in the sample was the real reason for the OER activity in this study. The XPS spectra of Co in sample CP3 (Figure 7a) showed main peaks at 780.0 eV (Co 2p_3/2_) and 795.1 eV (Co 2p_1/2_). After OER, in the high-resolution spectrum of Co 2p, detected only Co^3+^ was detected (two main peaks at 780.6 eV and 795.9 eV) (Figure 7d) [54]. While reviewing the above results, it can be inferred that the sample CP3 exhibited a higher electrocatalytic performance (during OER) compared to the rest of the samples. The boosted activity might be due to the several reasons, such as the electron structure effect and surface properties etc. It has been established that the Co_3_O_4_ and LiCoO_2_, are p-type semiconductors [14]. When a metal oxide electrode is inserted into an electrolyte, an electrical double layer or space charge layer is formed. For p-type semiconductors, the space charge layer is negligible [51]. So, as a catalyst material, a p-type semiconductor is more suitable. Secondly, the active sites in delithiated LiCoO_2_ are much more than LiCoO_2_ catalyst. With delithiation, the Co–O bond covalency increases which may lead to the development of holes in the hybridized Co 3d—O 2p orbitals, making the material more electrophilic aiding the adsorption of the hydroxyl group and hence enhancing the OER activity [16]. Furthermore, it is also known that electrochemical performance can be strongly influenced by the surface properties of the catalyst as the reactions usually occur on the surface of the catalyst. Figure 7a–f represents the XPS spectra of the CP3 sample after the OER stability test. As evident from the XPS, the sample CP3 was rich in Co^3+^ and O^−2^ species, both of which are electrophilic agents. In Figure 7e the binding energy value 531.9 eV associated with surface O_2_ species could be ascribed to non-stoichiometric oxygen, reported as the active site in OER catalyst, which occupied a higher percentage of 52.3% (=(non-stoichiometric oxygen)/[((non-stoichiometric oxygen) + (hydroxyl oxygen)). In comparison, 532.8 eV (with a proportion of 47.6%) is associated with chemisorbed or dissociated oxygen or the presence of OH species on the surface. Figure 7e shows a small shift in the binding energy in the O 1s spectrum from 531.9 eV to 531.68 eV. The peak at around 531.68 eV could be associated with non-stoichiometric oxygen, which occupied a higher percentage (57.6% compared with the 52.3% pre-OER test), and only a 5% reduction in the intensity of the 532.8 eV peak was observed after the OER. The increasing percentage of non-stoichiometric oxygen may be assigned to the transition from Co_3_O_4_ to CoOOH. The analysis results suggested that Co_3_O_4_ on the surface was transformed to CoOOH, which is in fact the active species to OER in some literature works. Moreover, electrochemical stability is another important criterion for measuring electrocatalysts’ performance; the stability of configured catalysts was investigated by chronopotentiometry (CP). A slight shift in the XPS results after the chronopotentiometry test proved the excellent stability of the CP3 sample for the OER catalyst [13].

## 4. Conclusions

Lemon peel extract along with citric acid proved to be a good reducing and leaching agent for the recovery of metals from waste lithium-ion batteries. Furthermore, the economical and environmentally friendly nature of the method may aid further in solving problems related to pollution. The subsequent recovered material after calcination at different temperatures was tested for OER activity and it was established that the recovered material calcined at 600 °C was not only best amongst all of the samples but was also stable in terms of catalytic activity. Its average particle size was also within 20–100 nm range and the BET surface area was calculated to be 4.8027 m^2^/g. A low potential of 0.55 V vs. RHE was required for the current density to reach 10 mA/cm^2^ with a Tafel value of 128 mV/dec.

## Figures and Tables

**Table 1:** BET surface area.

	BET Surface Area (m^2^/g)	Pore Size (nm)	Pore Volume (cm^3^/g)
CP1	16.3016	17.53716	0.071471
CP2	5.1648	10.98172	0.014216
CP3	4.8027	11.57406	0.013897
CP4	2.5255	6.68136	0.004218
